# Combining experiment and prediction to explore surface chemistry and dissolution

**DOI:** 10.1107/S2052252525001782

**Published:** 2025-02-27

**Authors:** Andrew G. P. Maloney

**Affiliations:** a The Cambridge Crystallographic Data Centre 12 Union Road CambridgeCB2 1EZ United Kingdom

**Keywords:** crystal surfaces, pharmaceuticals, particle shape, *CSD-Particle*, dissolution

## Abstract

A crystal structure is often an integral component in the development of a new pharmaceutical product, and these structures are frequently used to understand, assess and often predict both the manufacturing and *in vivo* behaviour of these compounds. Combining a range of analytical methods with computational analysis of the crystal surfaces, Zmeškalová *et al.* [(2025). *IUCrJ*, **12**, 141–154] link the properties of three solid forms of a biologically active molecule to its dissolution behaviour.

In this issue of *IUCrJ*, Zmeškalová *et al.* (2025 ▸) present a study linking the dissolution behaviour of three solid forms of a biologically active molecule, cannabigerol, to the structures and surface properties of these crystals. A variety of experimental analytical methods alongside computational studies of the crystal structures are used to explain the differences in behaviour that are observed between the solid forms. This article highlights the growing impact of computational tools to enable the digital design of new active pharmaceutical ingredients (APIs), while demonstrating the ongoing importance of crystallography and other experimental techniques to support these observations.

There are a multitude of challenges associated with bringing a new compound to market as a novel drug product (Torjesen, 2015 ▸), and natural products often represent an attractive alternative. However, as with many pharmaceuticals, the biopharmaceutical properties of these compounds, particularly their solubility, are often not conducive to easy uptake in the body (Amidon *et al.*, 1995 ▸). The use of alternative solid forms such as co-crystals, where the API is crystallized alongside another compound that is either benign or that can have an additional therapeutic effect, is a common strategy used to improve these critical properties (Bolla & Nangia, 2016 ▸).

During the development of a new drug, a wide range of different solid forms, including polymorphs, salts and co-crystals, are screened to assess their physical properties to ensure that the optimum form is selected for formulation and manufacturing. X-ray crystallography is often referred to as the ‘gold standard’ of techniques (Bond, 2014 ▸) that can be used to characterize the structures of these different forms, complemented by techniques such as differential scanning calorimetry (DSC) to understand their thermal properties and relationships between polymorphs. Repositories of crystallographic information such as the Cambridge Structural Database (CSD, Groom *et al.*, 2016 ▸) are routinely used to provide further context to understand the relative stabilities of different solid forms through informatics-based approaches (Feeder *et al.*, 2015 ▸).

The role of solid-form selection alongside efforts to engineer particles with favourable attributes has become key to the rational design of drug products (Ticehurst & Marziano, 2015 ▸). More recently, computational modelling or ‘digital design’ approaches have seen increasing use within the pharmaceutical industry (Abramov *et al.*, 2022 ▸), providing complementary analysis to established experimental techniques while increasing productivity and reducing the need for precious API material early in the drug development process. Of course, with all new digital tools, care must be taken to ensure that the findings are supported by experimental observations.

Cannabigerol is a member of the cannabinoid family which shows a variety of desirable pharmacological effects, such as anti-inflammatory properties, without some less desirable attributes – it is non-psychotropic. However, cannabigerol’s relatively low melting point and low solubility present challenges to delivering it to patients through common orally administered routes such as tablets or capsules. Zmeškalová and coworkers present a route to overcoming these challenges through co-crystallization of cannabigerol with piperazine, which is widely used in a variety of pharmaceutical formulations, and tetra­methyl­pyrazine, which is used in traditional Chinese medicine.

The authors use DSC to establish the thermal properties of the multicomponent materials, demonstrating that the new co-crystals both have higher melting points than pure cannabigerol. Low melting points can pose challenges during pharmaceutical processes, therefore the observed thermal stability of the co-crystals indicates that they may be more suitable for manufacturing. Experiments to measure the dissolution rate of cannabigerol and the two co-crystals show that while no significant increase is observed for the piperazine co-crystal, the solid form that contains tetra­methyl­pyrazine shows almost three times the dissolution rate of pure cannabigerol. Higher dissolution rates are an important factor in how quickly an API can be absorbed by the body.

Zmeškalová and coworkers then use single crystal X-ray diffraction to explore the structures of the three solid forms, highlighting differences in the molecular geometries, the way the molecules pack together in the solid state and the different ways in which they interact with each other. These observations are then combined with new computational tools from the Cambridge Crystallographic Data Centre’s *CSD-Particle* suite to rationalize the observed differences in the behaviour of these crystals in terms of their surface chemistry and topology. Using the newly reported crystal structures to predict the shape of each particle and comparing this prediction to what was observed experimentally, the authors then calculate the properties of the facet with the greatest surface area for each system. It is demonstrated that for its main surface the cannabigerol:tetra­methyl­pyrazine co-crystal displays a higher instance of polar functional groups as well as increased interactions with water molecules based on data from the CSD than the other two structures, correlating with the observed relative increase in dissolution rate. An example of this analysis is shown in Fig. 1 ▸.

The article by Zmeškalová and coworkers demonstrates the value of combined experimental and computational workflows to understand the impact of differences between solid forms on the properties of pharmaceutically relevant materials. While further studies are required to make such digital design approaches truly predictive, the conclusions of this work provide an important step along this path and highlight the value of structural analysis through crystallography in the pharmaceutical industry.

## Figures and Tables

**Figure 1:**
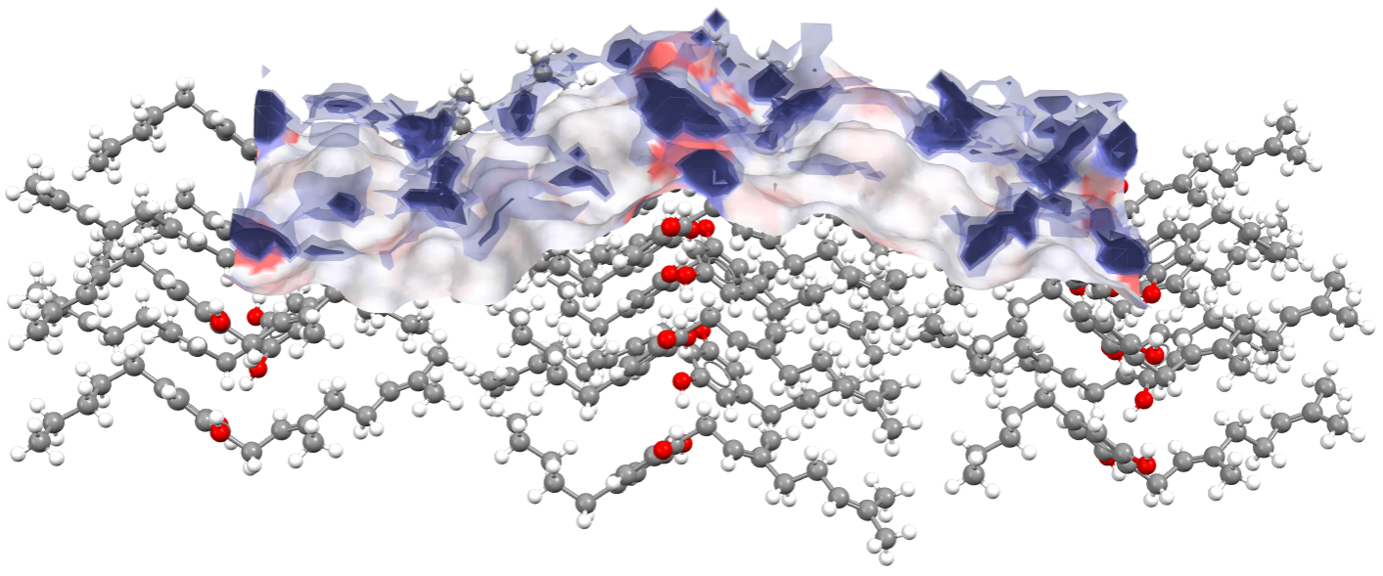
The interactions of water molecules (represented as blue clouds) around more polar areas of a predicted particle surface (shown by the red regions) are linked to dissolution behaviour by Zmeškalová and coworkers.
